# Development of an accurate low cost NDVI imaging system for assessing plant health

**DOI:** 10.1186/s13007-023-00981-8

**Published:** 2023-01-30

**Authors:** John D. Stamford, Silvere Vialet-Chabrand, Iain Cameron, Tracy Lawson

**Affiliations:** 1 School of Life Sciences, University of Essex, Colchester, CO4 3SQ Essex UK; 2 Present Address: Horticulture and Product Physiology, Department of Plant Sciences, Wageningen University & Research, 16, 6700 AA Wageningen, The Netherlands; 3 Environment Systems, 9 Cefn Llan Science Park, Aberystwyth, SY23 3AH Ceredigion UK

## Abstract

**Background:**

Spectral imaging is a key method for high throughput phenotyping that can be related to a large variety of biological parameters. The Normalised Difference Vegetation Index (NDVI), uses specific wavelengths to compare crop health and performance. Increasing the accessibility of spectral imaging systems through the development of small, low cost, and easy to use platforms will generalise its use for precision agriculture. We describe a method for using a dual camera system connected to a Raspberry Pi to produce NDVI imagery, referred to as *NDVIpi.* Spectral reference targets were used to calibrate images into values of reflectance, that are then used to calculated NDVI with improved accuracy compared with systems that use single references/standards.

**Results:**

*NDVIpi* imagery showed strong performance against standard spectrometry, as an accurate measurement of leaf NDVI. The *NDVIpi* was also compared to a relatively more expensive commercial camera (Micasense RedEdge), with both cameras having a comparable performance in measuring NDVI. There were differences between the NDVI values of the *NDVIpi* and the RedEdge, which could be attributed to the measurement of different wavelengths for use in the NDVI calculation by each camera. Subsequently, the wavelengths used by the *NDVIpi* show greater sensitivity to changes in chlorophyll content than the RedEdge.

**Conclusion:**

We present a methodology for a Raspberry Pi based NDVI imaging system that utilizes low cost, off-the-shelf components, and a robust multi-reference calibration protocols that provides accurate NDVI measurements. When compared with a commercial system, comparable NDVI values were obtained, despite the fact that our system was a fraction of the cost. Our results also highlight the importance of the choice of red wavelengths in the calculation of NDVI, which resulted in differences in sensitivity between camera systems.

**Supplementary Information:**

The online version contains supplementary material available at 10.1186/s13007-023-00981-8.

## Background

To meet the demands of an increasing population and to maintain food security, production of new varieties and cultivars of crops with increased yields and/or the ability to cope with predicted changes in climate, will be required. In order to produce these new varieties, intensive crop breeding programmes are key. However, currently the rate at which new varieties with improved performance can be screened for phenotypic traits of interest is outpaced by the rate at which new varieties can be produced and genetically screened [1]. Spectral imaging (a technique that uses single or multiple wavebands within the electromagnetic spectrum to provide an indicator of a desired trait) has greatly assisted in overcoming this bottleneck, by providing rapid, non-invasive and in situ measurements on relatively large numbers of plants or crop canopies. This is achieved through the measurement of reflected light, which is affected by the physical and biochemical properties of a leaf. Although spectral images are often relatively easy to capture, the necessary expertise to process these images and interpret the outputs has restricted its use [1–3]

The Normalised Difference Vegetation Index (NDVI) is a measure of the ratio of reflectance in the near infra-red (NIR) and red wavebands [4, 5] and is one of the most frequently used spectral indices in both research and agriculture as a rapid and easy method to detect vegetation and assess overall plant health [6]. The measurement is based on the principle that the cell structure of a leaf strongly reflects NIR due to a lack of absorption by plant pigments, while chlorophyll pigments strongly absorb red wavelengths. Plants that are ‘healthy’ with high chlorophyll content absorb more red and therefore reflect a higher proportion of NIR than ‘less healthy’ plants. NDVI is determined using the following formula, with NIR and red being reflectance values that vary between 0 and 100%.

1 $$\begin{array}{c}NDVI= \frac{\left(NIR\,-\,RED\right)}{\left(NIR\,+\,RED\right)}\end{array}$$

NDVI values are normalised to range from -1 to 1, with positive values indicating more NIR than red reflectance. For healthy vegetation, there will be a greater relative absorption of red by chlorophyll compared to NIR, and NDVI values will approach 1. As chlorophyll levels diminish due to stress or senescence, NDVI will approach 0 due to less absorption of visible red light [7].

It should be noted that, despite the prevalence of NDVI as a measurement, there is no universally agreed-upon standard for the specific red and NIR wavelengths that should be used to determine NDVI, and although several optimal wavelengths have been proposed [7], it is common to see literature citing the use of many different wavelengths [8, 9]. However, the choice of waveband(s) often depends on the technical capability of the equipment, for example the number of specific bands available and the bandwidth of the sensor [10, 11].

The availability of NDVI imaging systems has increased alongside the increasing popularity of unmanned aerial vehicles (UAVs), and while easy to use and readily available, commercial systems can be expensive, with prices typically ranging from USD$2000 up to USD$5000 per device. Commercial systems often include the additional involvement of deploying UAVs or integrating expensive imaging systems onto agricultural machinery, adding to the overall cost of equipment/application [12]. Optical satellite images which provide NDVI are available at no cost to users from platforms such as the ESA Sentinel-2 or NASA Landsat-8 satellites. These datasets are used to measure NDVI for agriculture and ecology, but provide relatively lower resolutions of 10 × 10 m for Sentinel-2 and 30 × 30 m for Landsat-8 [13, 14]. Higher resolution satellite NDVI imagery is also available from a variety of commercial providers, with per-scene costs typically in the range of hundreds to thousands of dollars. There are a number of more affordable non-commercial devices available (Table 1), with a popular choice often being consumer level, digital camera based systems often priced at around USD$380 (at the time of publication) that can capture NDVI imagery using NIR and blue wavelengths. However, systems that employ the use of blue wavebands (instead of red) for determining NDVI type measurements come with some drawback. Reflected blue wavelengths are less sensitive to changes in chlorophyll content compared with red wavelengths [15] because of high absorption in the blue region by plant pigments [16].

**Table 1:** A comparison of different low-cost NDVI camera systems, highlighting the pros/cons of each system, and indicating the main advantage of the NDVIpi system described here

References	Approx. price (camera, filter and controllers) (GBP)	Pros	Cons	Advantage of NDVIpi
[59]	£360	Two-camera seperation of Red/NIR; off-the-shelf consumer cameras; good quality optical filter	2 or Less references	NDVIpi uses 6 references to account for non-linearity of consumer cameras; Lower-cost optical filter is used without detriment to accuracy
[60]	£594 (excluding optical filters)	Two-camera seperation of Red/NIR; off-the-shelf consumer cameras; good quality optical filter; doesn't require calibration targets; vignetting correction	Requires on-site downwelling spectrometer	Programmable camera control; low-cost optical filter; can be customised and integrated with other systems; affordable reference targets
[47]	£545	Dual-band optical filter on a single camera; multiple references	No programmable control of camera, not easy to integrate with other systems	Programmable camera control; low-cost optical filter; can be customised and integrated with other systems
[46]	£100 (excluding filters)	Two versions, one with a low-cost optical filter and one with a dual-pass optical filter; multiple references; single camera (no need for alignment)	No programmable control of camera. Leakage of NIR and Red (low-cost optical filter) due to single camera	Programmable camera control; low-cost optical filter; can be customised and integrated with other systems; low-cost filter; dual-camera to present NIR leakage
[18]	£110	Multiple References; Calibration to account for non-linearity; Two-camera seperation of Red/NIR	Requires camera modification	Programmable camera control; low-cost optical filter; can be customised and integrated with other systems; requires assembly
[19]	–	Two-camera seperation of NIR and red; good quality optical filters	No calibration	Calibration methodology and multiple references; Lower-cost optical filter is used without detriment to accuracy
[61]	£325 (£406 for reference targets)	Multiple References; Calibration to account for non-linearity; One camera with high quality optical filters		Programmable camera control; low-cost optical filter; low-cost reference targets; can be customised and integrated with other systems; calibration inherently accounts for gamma; correction (thus any camera can easily be used in the NDVIpi without requiring determination of the gamma correction)
[62]	£200 (excl. filters)	Two-camera seperation of NIR and red; good quality optical filters	Requires on-site downwelling spectrometer	Calibration methodology and multiple references; Lower-cost optical filter is used without detriment to accuracy; an be customised and integrated with other systems
[63]	£60 (excl. filters)	Raspberry Pi based; good quality optical filters; single camera system	2 or Less references	NDVIpi uses 6 references to account for non-linearity of consumer cameras; Lower-cost optical filter is used without detriment to accuracy
[48]	£785	Good quality optical filters; single camera system	2 or Less references	Low-cost reference targets; can be customised and integrated with other systems; calibration inherently accounts for gamma; correction (thus any camera can easily be used in the NDVIpi without requiring determination of the gamma correction)
[12]	£968	Narrowband filters over CCD camera; Good quality optics; Two camera seperation; Very similar to commercial cameras		Off-the-shelf components
[64, 65]	~ £200	Low-cost filters	Blue channel used for visible wavelengths; no hard seperation of VIS/NIR	Red wavelengths for NDVI calculation; two camera seperation; Programmable camera control; Six references
[22]	£712	Single Camera; No optical filters; algorithmically seperates NIR and visible images		Lower cost

The approximate price point for all of these cameras has been estimated, but actual prices may vary. The price only accounts for the purchase of camera(s), filters, and any computer based controllers (e.g. Raspberry Pi, Arduino, etc.) that were used to capture imagery. The price was not been included for one system due to the difficulty in obtaining a reasonable estimate for the equipment that was used

There are methodologies and technologies available for developing NDVI imaging instruments, and in many cases these are aimed at providing a low cost solution for research, environmental monitoring, and agriculture (Table 2) [12, 17, 18]. One common approach is to use two separate cameras to capture the two required wavebands: one RGB camera with the infra-red (IR) blocking filter removed and replaced with a narrow band NIR filter (e.g. 700–800 nm), which effectively converts the red colour channel into an NIR sensor [18–21]. A second RGB camera captures red wavebands using the red channel, and the two datasets used to construct images of NDVI. The construction of many of these systems requires time and skill to setup, and the cost of the cameras and optical filters can be high. Additionally, a two camera approach requires image alignment of the two images for calculation of NDVI which introduces a further complexity [12, 17]. However, these systems show good linear relationships to NDVI measured spectrometrically or via satellite [20].

**Table 2:** Camera settings for the two cameras used for the Raspberry Pi Imaging system

Camera setting	*NoIR PiCamera*	*PiCamera (RGB)*
ISO	400	400
Shutter Speed (µs)	2500	400

Other strategies to measure NDVI include instruments designed to implement custom dual band pass optical filters, which allow narrow regions in red and NIR wavelengths to pass to a single camera sensor [17]. This enables measurements of red and NIR by a single device, thereby enabling both images to be collected simultaneously and removing some of the complexity of downstream image alignment (outlined above). A similar approach is used in commercial systems, such as the UAV based cameras built by Sentera (Minneapolis, MN, USA) and AgroCam (Debrecen, Hungary). One creative approach has been to use a web cam based security camera to derive visible and NIR imagery from a single camera, exploiting the night vision feature of such cameras [22]. There are many examples of camera systems designed for environmental monitoring, tracking relative changes in NDVI and greenness throughout the seasons [23–25]. A number of low-cost camera systems listed are available (Table 1), with the majority utilizing consumer level digital cameras, with the issues already discussed above. Furthermore, these cameras are not easy to programme, customise or integrate with other systems (e.g. UAV control, phenotyping platforms, etc.). Also, the non-linearity of these cameras is often not accounted for. Although these systems are typically considered ‘low-cost’ they can still be quite expensive, with 5 out of 13 listed in Table 1 in excess of GBP£500, and only 5 systems with a price point below £400. In Table 1 we have highlighted the key advantages and drawbacks of these devices and compared them with the system we describe here.

However, many previously developed imaging systems lack a robust reflectance calibration that is essential to generate accurate NDVI values, especially accounting for the non-linearity of off-the-shelf cameras. Likewise, many systems are not readily customisable or easy to integrate with other platforms. Here we describe the development of an alternative NDVI imaging system based on the affordable off-the-shelf Raspberry Pi and the NoIR camera, lowering the barrier of entry for many users. The Raspberry Pi is a small single board computer, initially designed as an educational tool, but has since found popularity from hobbyists to researchers across a number of disciplines, and more importantly is priced affordably and offers considerable flexibility and customisation [26]. When coupled with suitable cameras, the Raspberry Pi can be used for image capture and has previously been used in plant imaging; for measuring leaf area [27], plant shape, height and other physical traits [28], all of which are valuable traits for phenotyping for high biomass crops. The release of a camera with no NIR filter for the Raspberry Pi (NoIR Camera) has opened new possibilities to develop an affordable and accurate NDVI sensor, which could be used by scientists and the public. However, for such a system to deliver accurate and reliable measurements between experiment requires reflectance calibration. The easiest calibration method, the empirical line method [29], uses 2 known reflectance reference materials with high and low values and assumes a linear change in reflectance between the two standards. However, due to the non-linearity of off-the-shelf cameras, this assumption does not always hold true, therefore, we propose the use of 6 reference standards that takes into account the non-linear relationship between the camera signal and the reflectance measurements. This unique calibration approaches improves the accuracy and robustness of reflectance estimations and therefore values of NDVI.

In this manuscript, we outline the design, construction and calibration of our low cost *NDVIpi* system and provide details of methodology for imaging processing to ensure high quality accurate NDVI imagery without substantial expertise required.

The system described here brings together many key physical and operational features that to date have rarely been combined into a single system, and outline a methodology that improves the accuracy of measurements, ease-of-use and customisation. These features include; use of the Raspberry Pi platform with low-cost cameras; inexpensive colour filters for NIR imagery, dual-camera separation of visible and NIR imagery, simple robust calibration using six reference standards to account for non-linearity of camera, and image alignment without the need of commercial software, and computer control, which can be customised and extended as required by the end-user. Although the system described here has been specifically developed to measure NDVI, it can also serve as a platform that could be modified by the user to extend to measure other spectral indices or spectral regions on interest using the same procedures described here. The approach and methodologies outlined in this paper could also be applied to other camera systems.

## Materials and methods

### Setting up the Raspberry Pi for NDVI imagery, the “*NDVIPi*”

**Fig. 1:**
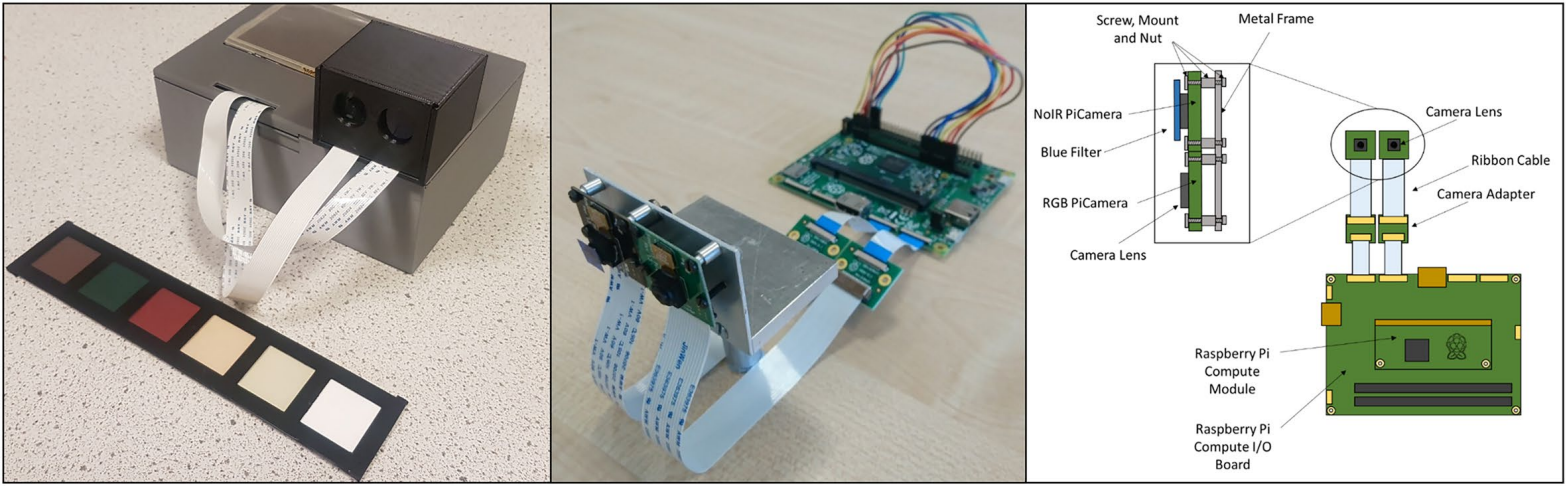
An image and schematic of the core setup for the Raspberry Pi Compute, PiCamera and NoIR PiCamera, and a metal camera holder. The camera holder keeps the two cameras at the same plane, allowing for good alignment of captured images

To construct the *NDVIPi* system, the Raspberry Pi Compute Module (Raspberry Pi Foundation, Caldecote, UK) [30] was used in conjunction with the Raspberry Pi I/O Board. The I/O board has two individual camera ports (Fig. 1), which enables the attachment of two cameras to the Raspberry Pi via the Raspberry Pi CMDK Camera Adapter. The first was a standard RGB camera designed for the Raspberry Pi, known as the PiCamera and was used to capture visible wavebands on the red channel. The other was an off-the-shelf NoIR PiCamera which has no Infra-Red (IR) blocking filter, meaning that all channels also capture NIR light. Both cameras come from the manufacturer with an Omnivision OV5647 CCD sensor (Omnivision, California, USA) and images were captured in an unencoded RGB format. An Alice Blue 197 blue plastic filter (Lee Filters, Hampshire, UK) was placed over the lens of the NoIR camera (Fig. 1). The filter had a low transmittance of red wavelengths (Additional file 1: Fig. S1), while allowing infra-red light through. Although both cameras measure light on all of the three channels; Red, Blue and Green (RGB), only the red channel from each camera was used, providing the visible red image from the RGB camera, and the NIR image from the NoIR camera. However, as the Alice Blue filter is not a blocking filter and allows transmission of a small amount (5–15%) of red wavebands, the red channel of the NoIR camera (intended for measuring only NIR), captures a small amount of red light. This can be accounted for by calculating the % of light that leaks through the filter and removing this fraction from the intensity of the NIR imagery (See Additional file 1: Fig. S2). Additionally, it should be noted that the red channel of both cameras captures a small amount of blue and green light. Based on the general broad spectrum of a typical Bayer filter [31–33] the central wavelength selected for the visible red channel was 620 nm, while the central NIR wavelength was selected for 750 nm. The physical properties of the PiCamera and NoIR, such as the effect of camera vignetting and how to correct for it, has previously been discussed in detail within the literature [33, 34].

A camera holder was designed and constructed to allow side-by-side stereo placement of the cameras (Fig. 1), which provided a way to align the two camera boards and assisted in improving the accuracy of image alignment of the two images. Camera settings used for both cameras are listed in Table 2. The non-filtered RGB PiCamera was prone to saturation and thus the shutter speed was often lower than the NoIR camera. It was essential to adjust and configure shutter speed and ISO (International Organisation for Standardisation) sensor settings to take into account the light environment, to ensure all images utilised the cameras full sensor range. Shutter speed was determined by taking a series of photos across a range of shutter speeds in situ. Images were subsequently analysed using the Fiji/ImageJ software [34] to determine the highest shutter speed in which images from the visible red channel were not saturated. We opted for shutter speeds in which the brightest reference (see section below) had a digital number below 240. This step is essential, as it is not possible to accurately calibrate over the full reflectance range (0–100%) if images are saturated.

### Calibration of *NDVIpi* and *Micasense RedEdge* images

#### Image calibration and selection of reference materials

All *NDVIpi* code, image manipulation and image calibration was performed using the Python language (Python Software Foundation), OpenCV library (opencv.org) and NumPy (numpy.org). Image alignment and general image analysis was performed with the Fiji/ImageJ software [34]. Image alignment was also performed using Python and feature based detection libraries as part of the OpenCV library using the ORB algorithm [35]. Image alignment algorithms detect similar features in both NIR and red images, and then transform the NIR image to overlay the features of the NIR image as closely as possible to the features of the red image.

**Fig. 2:**
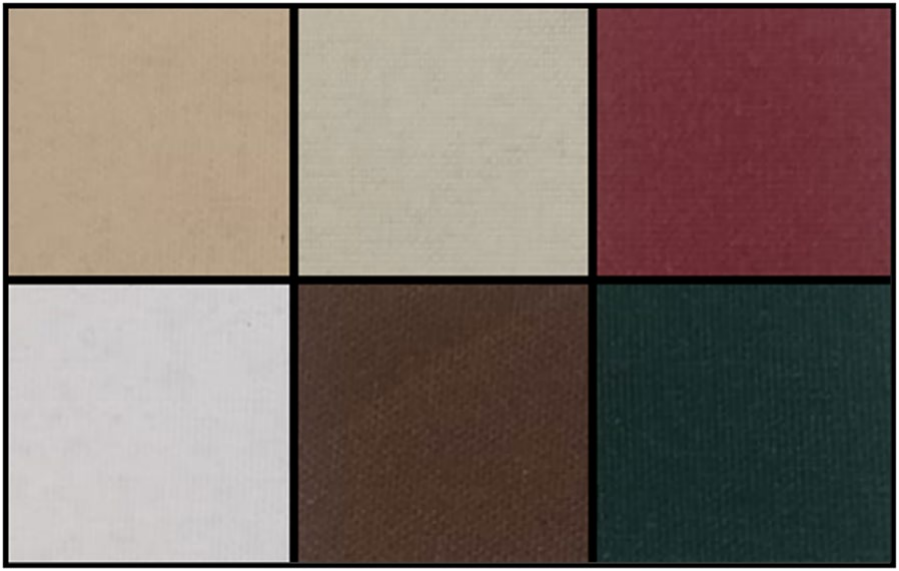
Raspberry Pi Calibration board, consisting of six diffuse reference materials with known relative reflectances (Table 3 ), which are used to calibrate the digital number of images captured by the Raspberry Pi to actual measurements of relative reflectance

Images were calibrated using a version of the empirical line method [29] which used six materials of known reflectance to convert images from digital (‘pixel’) numbers into reflectance. Calibration of the *NDVIpi* was performed using an in-house developed calibration board, consisting of squares (3 cm^2^) of six Kayospruce Odyssey material (Kayospruce, Hampshire, UK) with a range of reflectance values in the red and NIR wavelengths fixed to a flat hard backing (Fig. 2). The material was selected because of robustness, UV resistance, spectral consistency and relatively good diffuse properties. However, it should be noted that any materials with known spectral reflectance can be used for calibration. This calibration board was used in all subsequent image capture for independent calibration. However, for outside imagery under a clear sky or in an indoor environment with consistent lighting (e.g. a phenotyping platform), instead a single picture can be taken of the calibration board which can be used to calibrate subsequent images using the values of this initial image.

**Fig. 3:**
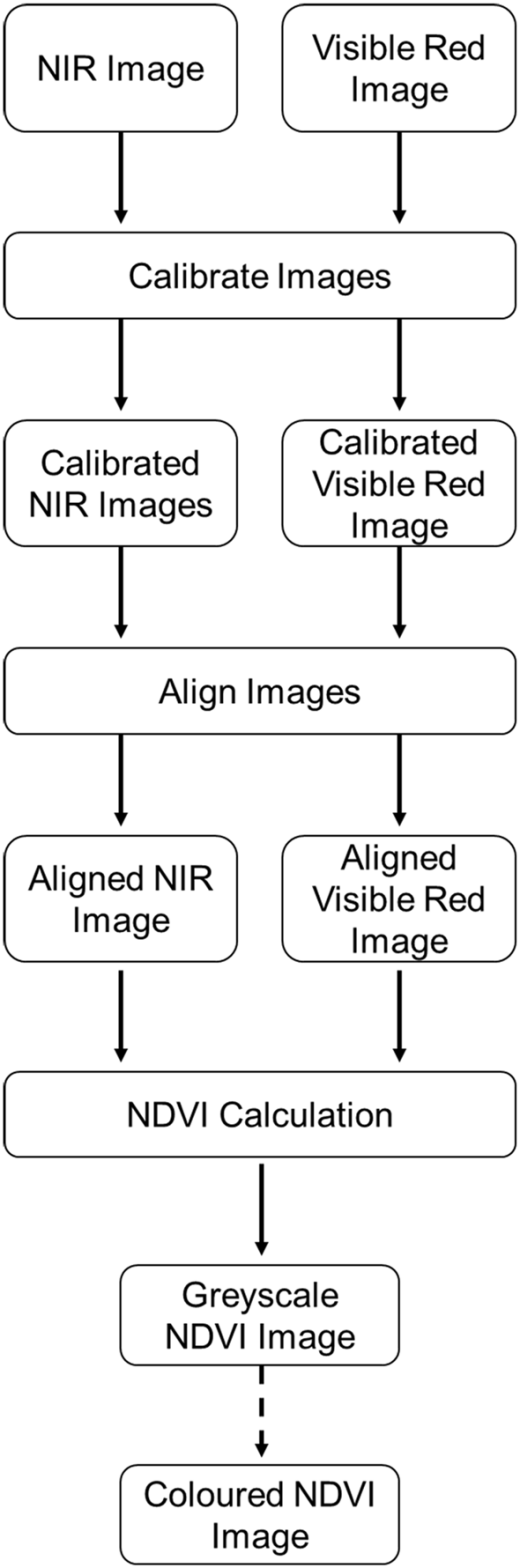
The process of calibration, showing each step from captured images to the generation of the output NDVI image. Dashed arrows show an optional step

**Fig. 4:**
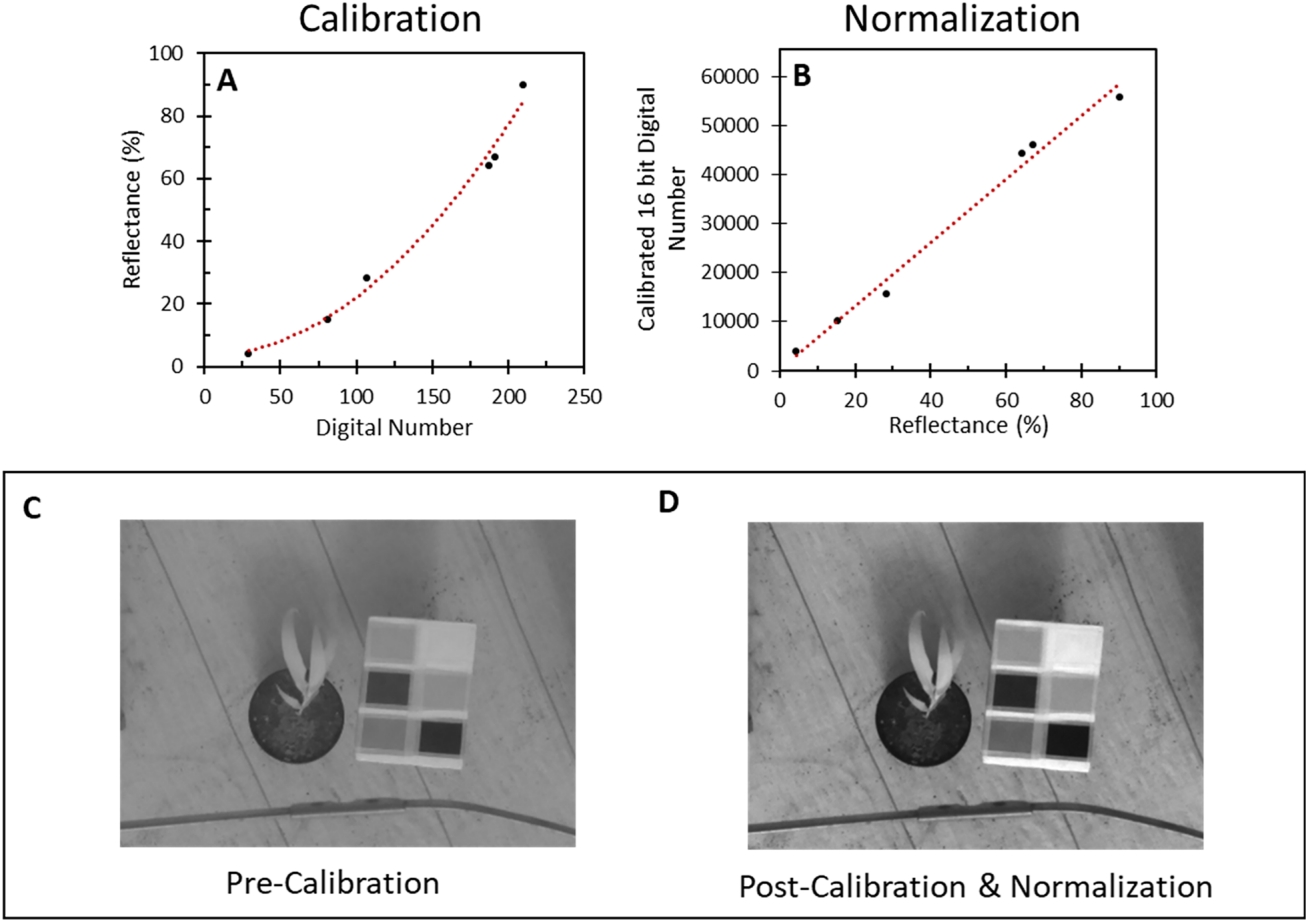
Example data showing calibration and normalization. **A** Calibration of raw digital number, direct from images captured by a camera, using the known reflectances of the six calibration materials to find the relationship between digital number and reflectance. Once the relationship is established for an image, raw digital number for the entire image can be converted into values of reflectance. **B** Normalization of reflectance data, by re-scaling the converted reflectance values (from 0 to 100%) to use the full 16 bit (0–65,535) range. **C**, **D** A typical NIR image, demonstrating the visual difference between images before **(C)** and after **(D)** the calibration and normalization process, with the image on the right now using the full range of the image, with image colour (from white to black) corresponding to actual values of reflectance

To perform the calibration (Fig. 3), for each image the average digital number (ranging from 0 to 255) of each of the six calibration materials was determined for both the red channel from the RGB camera, and the red (‘NIR’) channel from the NoIR camera. The relationship between the measured digital number of the calibration material, and the known reflectance (Table 3; Additional file 1: Fig. S3) was determined (Fig. 4A). Using this relationship, the digital numbers for each individual pixel across an entire captured image was converted into reflectance values using this relationship (Fig. 4A). The relationship between spectral reflectance and pixel value in Fig. 4A were not entirely linear, due to the effect of gamma correction. This is a technique built into the hardware of the camera that modifies the brightness of an image to adapt it to the way human eyes perceive light and colour, and is applied by default in many off-the-shelf cameras such as the PiCamera, and as a result a transformation was applied to linearise the relationship. The second key step (Fig. 3) was to generate calibrated images by converting reflectance values back into an image format. Spectral reflectance values were scaled into unsigned 16-bit integers, with reflectances of 0–100% linearly assigned to values of 0–65,536 in the output (Fig. 4B, C). Out of bound values, i.e. those below 0% or above 100%, were assigned to 0 and 65,536 respectively. This scaling is a compromise between preserving radiometric resolution and reducing file sizes.

**Table 3:** Measured relative reflectance of the six reference materials of the Raspberry Pi calibration board, of Red (620–680 nm) and NIR (720–750 nm) light, derived from Additional file 1: Fig. S3

# (Colour)	Red reflectance (%)	NIR reflectance (%)
1 (*White*)	92.69	87.62
2 (*Sand*)	63.59	60.85
3 (*Brown*)	16.29	16.13
4 (*Indian Burch*)	66.72	62.69
5 (*Forest Green*)	4.90	5.88
6 (*Burgundy)*	25.53	39.40

Once saved as 16 bit PNG images, the calibrated images were loaded into a custom made python script which performed feature based image alignment as part of the OpenCV package and this step aligned the NIR and red images as closely as possible.

To generate output NDVI imagery (Fig. 3), the calibrated and aligned image pairs were processed using the OpenCV library to calculate NDVI pixel by pixel (following Eq. 2). The output of the NDVI calculation was then linearly scaled to 16-bits, with NDVI values of − 1 to + 1 assigned to 0–65,536 respectively. The greyscale image could then be coloured as desired, for instance by using the look up table (LUT) feature of software such as Fiji/ImageJ.

Thus, NDVI for the *NDVIpi* system was calculated as;

2 $$\begin{array}{c}{NDVI}_{RaspPi}= \frac{\left(R750\,-\,R620\right)}{\left(R750\,-\,R620\right)}\end{array}$$

With *R* denoting reflectance at a specific wavelength.

To rescale the NDVI image back to values within a range of − 1 to + 1, the digital numbers (0–65,536) in these areas were converted back into NDVI (− 1 to + 1), using Eq. 3, in which *Pixel* is the average digital number of the region of interest:

3 $$\begin{array}{c}NDVI=\left(Pixel\times \left(\frac{2}{65536}\right)\right)-1\end{array}$$

#### Micasense Rededge

To evaluate our in-house device (the “*NDVIpi*”) with a commercial NDVI imaging system, a *Micasense RedEdge* camera (Micasense, Seattle, WA, USA) was setup to collect pictures alongside the *NDVIpi* system.

The Micasense RedEdge (‘Micasense’) is composed of five narrowband cameras, and an automatic gain/exposure feature to prevent saturation. One of the cameras, the “Red” camera, has a wavelength of 668 nm with a full width at half maximum (FWHM) of 10 nm. The “Red Edge” camera has a wavelength of 717 nm with a FWHM of 10 nm. The “NIR” camera has a wavelength of 840 nm and a FWHM of 40 nm. Since the Micasense contains two separate NIR bands, the Micasense can calculate two separate NDVI indices. Thus, NDVI was calculated using either the NIR (840 nm) and Red Edge (717 nm) bands, with R denoting reflectance centred at a specific wavelength:

4 $$\begin{array}{c}{NDVI}_{Micasense1}= \frac{\left(R717\,-\,R668\right)}{\left(R717\,-\,R668\right)}\end{array}$$

5 $$\begin{array}{c}{NDVI}_{Micasense2}= \frac{\left(R840\,-\,R668\right)}{\left(R840\,-\,R668\right)}\end{array}$$

The Micasense RedEdge was calibrated using the exact same process as the *NDVIpi,* using all six references, however the Micasense RedEdge does not apply a gamma correction to images and therefore there was a linear relationship between measured digital number and spectral reflectance of the calibration board, so no transformation was applied to linearise the data.

### Plants and growth conditions

*Phaseolus vulgaris* (French Bean) were grown in a growth cabinet at 200 µmol m^−2^ s^−1^ of light. Wheat (*T. aestivum*) and barley (*H. vulgare*) plants were grown in a greenhouse under ambient lighting, with a lighting system providing 12 h supplementary lighting. Illumination for supplementary lighting was approximately 200 µmol m^−2^ s^−1^ and provided by sodium vapour lamps. French bean were grown in pearlite growing medium (Pearlite Standard, Sinclair Pro, Cheshire, UK), with half of the plants supplemented with Hoaglands solution [36].

### Reflectance measurements

Spectral reflectance measurements were collected with a FLAME-S Spectrometer (Ocean Optics, USA), and a Reflection Probe fibre optic (Ocean Optics, USA). A tungsten bulb provided illumination of 198 μmol m^−2^ s^−1^ through the central fibre optic of the probe, which is surrounded by a ring of fibre optics to collect the reflected light and feed this light to the spectrometer for measurement. A leaf clip was constructed to enable a fixed geometry between the probe end of the fibre optic and the plane of the sample, which was sprayed with matt black paint to reduce reflection within the leaf clip. A Spectralon reflectance standard (WS-1, Ocean Optics, USA), which has 99% diffuse reflection across the 400 to 1500 nm wavelength range, was used as a reference for 100% reflection.

### NDVI measurements under controlled irradiance

Plants of French bean*,* wheat and barley were measured under a known irradiance of 800 µmol m^−2^ s^−1^ supplied by an LED light source (Heliospectra AB, Göteborg, Sweden). For each measurement, a leaf of each plant was laid across a flat surface, approximately 60 cm below the LED light source. The calibration board was placed in the same plane as the leaf measurements and was present in all images. Directly after each image capture, spectral reflectance measurements were also taken from four quadrants of each *Phaseolus* leaf, or along the length of the leaf blade for wheat and barley. The two *NDVIpi* cameras were located above the leaves, facing down towards the leaf samples.

To verify the calibration methodology, NDVI values measured using the *NDVIpi* camera were compared with a range of NDVI values measured by a spectrometer (used as a standard). Initially values were calculated from the spectrometer using a fixed NIR wavelength (750 nm) and the red wavelength allowed to vary across the red wavebands (600–700 nm) until a value was obtained that matched closest to the image NDVI value. This was repeated using a fixed red wavelength (620 nm) and NIR allowed to vary (700–800 nm). The % difference between the NDVI of *NDVIpi,* and the array of spectrometer NDVI values calculated with varied wavelengths, was determined.

### Comparison of NDVI from the *NDVIpi* and *Micasense RedEdge*, measuring plants under ambient lighting in a greenhouse environment

The performance of the *NDVIpi* Imaging system was compared to the *Micasense RedEdge* by relating NDVI measurements from both instruments to NDVI values derived from spectrometry. Measurements were performed on *Phaseolus vulgaris* plants in a glasshouse under ambient lighting conditions in the greenhouse, ranging from 200 up to 900 µmol m^−2^ s^−1^. The *NDVIpi* and a *Micasense RedEdge* camera were elevated above the plants at a distance of 1 m. Images were taken by the *Micasense RedEdge* approximately 30 s after Raspberry Pi images were taken. The calibration targets were present in all images and collected by both camera systems. The coefficient of variation was calculated as standard deviation divided by the mean, and was used to assess the variability of NDVI measurements between the two systems.

## Results

### NDVI Measurements under defined irradiance

**Fig. 5:**
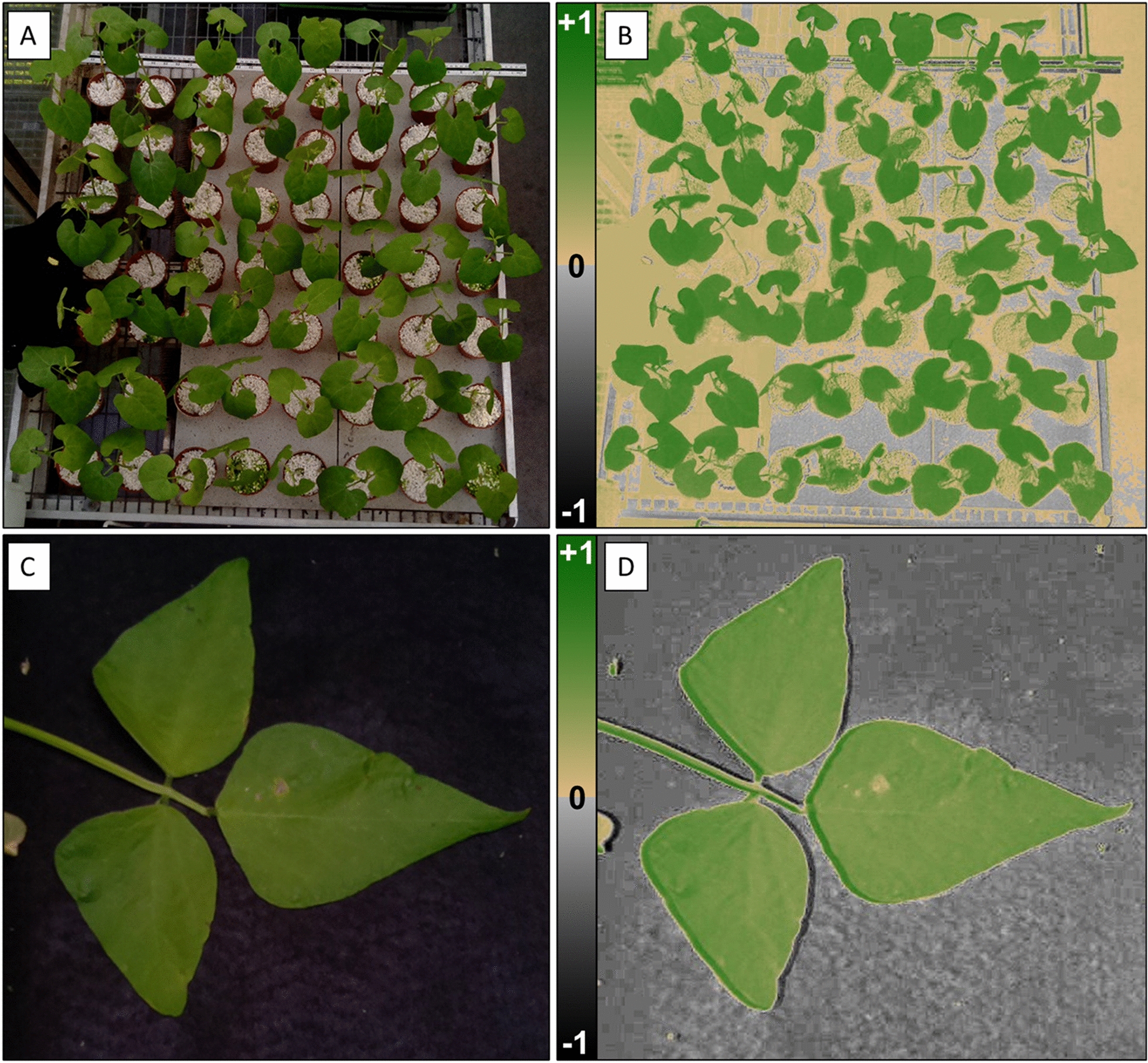
**A** RGB image of *Phaseolus* plants inside of a greenhouse. **B** Resulting coloured NDVI image of these plants. **C** RGB image of *Phaseolus* leaves taken under a controlled light source. **D** Resulting NDVI image. The colour scheme chosen produced an image that visually corresponds to the RGB image

**Fig. 6:**
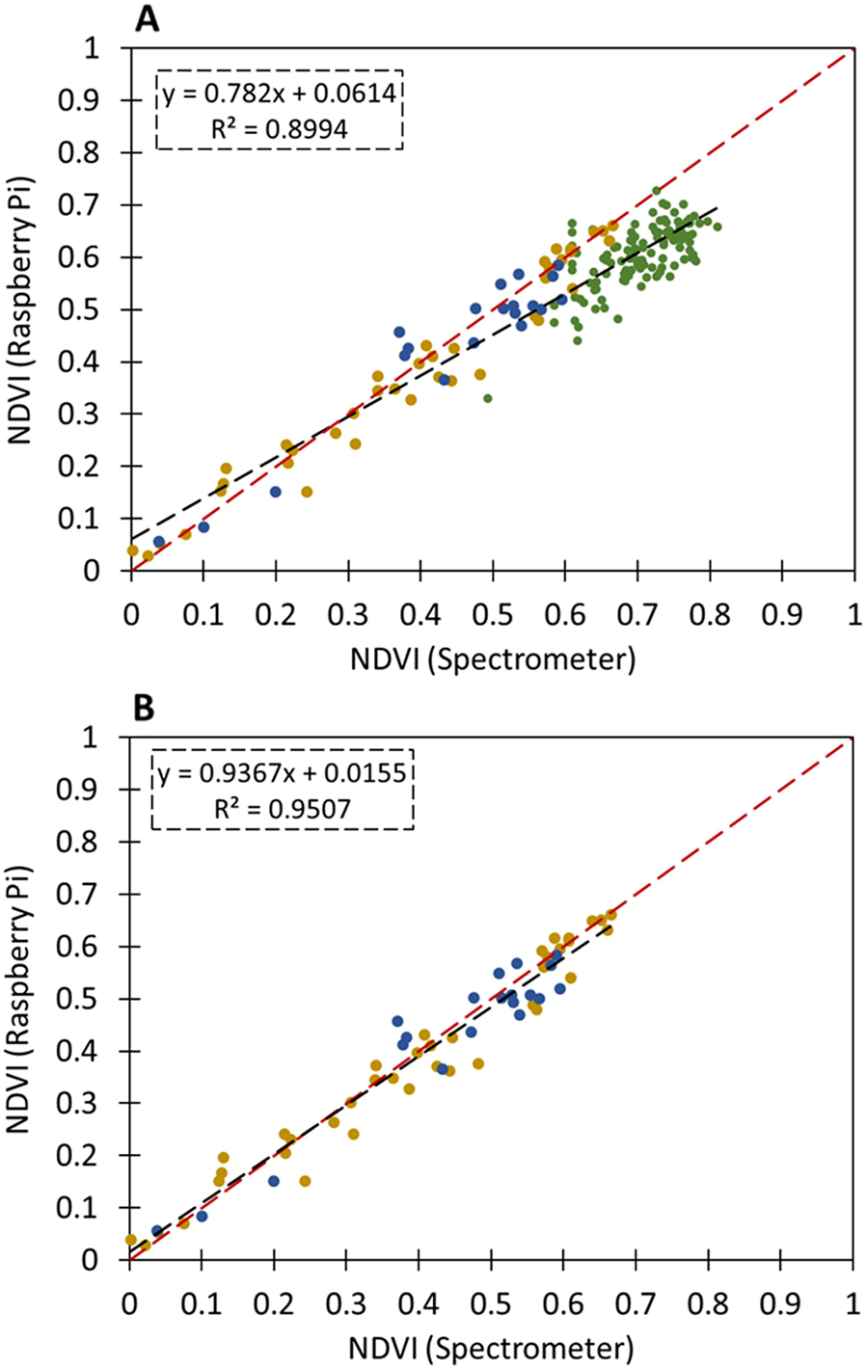
Comparison of NDVI imaging with the Raspberry Pi, and NDVI as measured with a spectrometer. Red dashed line represents a 1:1 relationship with NDVI calculated from the spectrometer. Wheat (Yellow), Barley (Blue), and Phaseolus (Green). **A** NDVI imaging compared to spectrometry NDVI (n = 181). **B** Dataset with *Phaseolus* measurements omitted (n = 59). In all instances, the Raspberry Pi images demonstrate a good relationship (R^2^ > 0.89) with spectrometry derived NDVI, highlighting the robustness of the system

Example images produced by the *NDVIpi* for *P. vulgaris* can be seen in Fig. 5. A colour scheme for NDVI was applied that produced an image that is visually comparable to the original RGB image (Fig. 5C, D), although a small halo around the leaf is visible that shows a slight mis-alignment of the images (Fig. 5D). To ensure that the images of NDVI produced by the *NDVIpi* system were accurate and provided a robust calibrated measure of NDVI, we verified the values with those derived from spectrometry and showed a strong positive relationship between spectrometry and *NDVIpi* values (Fig. 6) for all three plant species (R^2^ = 0.90), although NDVI values in the higher regions appeared to be underestimated with the *NDVIpi* (Fig. 6A). The large bulk of data in the high (> 0.6) NDVI region where predominantly from measurements on *Phaseolus* and omission of these data (Fig. 6B) improved the relationship (R^2^ = 0.95). Analysis of image histogram data, using 40 selected images of *Phaseolus* and wheat data, showed a greater skew towards a lower NDVI for the broad leaf *Phaseolus* (Skewness = 1.16, S.D. = 0.49) compared with grass leaves (Skewness = 0.67, S.D. = 0.47) (Additional file 1: Fig. S4).

### Verification of *NDVIpi* with spectrometer calculations

**Fig. 7:**
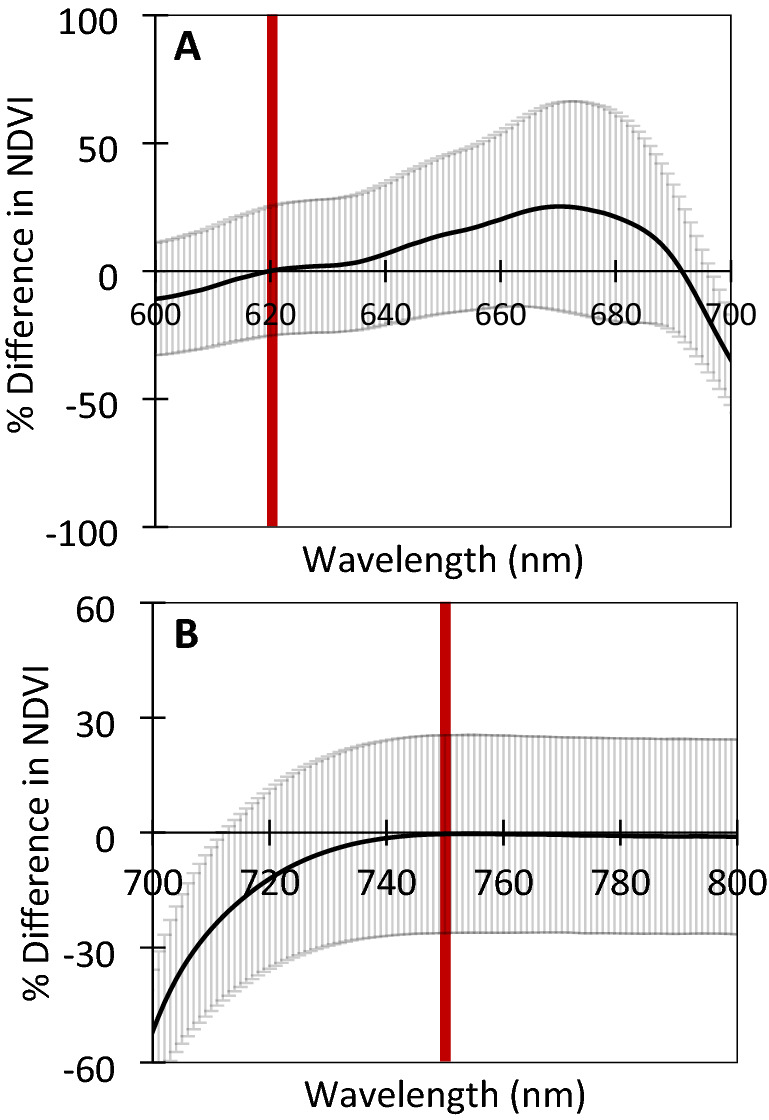
Analysis to verify calibration by testing an array of wavelengths from spectrometry-derived NDVI calculations, to identify which wavelengths produce NDVI values that most closely correspond to the NDVI values that were actually obtained from Raspberry Pi imagery. Measuring the percentage (%) difference between the two NDVI values. **A** NDVI with the wavelength for visible red ranging from 600 up to 700 nm, with NIR wavelength fixed at 750 nm. **B** NDVI wavelength for NIR ranging from 700 up to 800 nm, with visible red fixed at 620 nm. From this data, the wavelengths which produced an NDVI value that best matched the values as measured by the Raspberry Pi system, correspond to the wavelengths that the Raspberry Pi images were calibrated to; 620 nm for red and 750 nm for NIR, indicated by the red bars. Error ± S.D (n = 150)

By comparing the resulting NDVI imagery, calibrated to 620 nm for red and 750 nm for NIR, against an array of NDVI values measured by spectrometry which used a range of different wavelengths for both red and NIR in the NDVI calculation, verification of the calibration protocol could be appraised by determining if NDVI images produced the same values as the spectrometer using the same wavelengths, and if not, which combination of red and NIR wavelengths would generate the same NDVI data as the NDVI imagery. This verification showed that the images were indeed calibrated to a red wavelength of 620 nm (Fig. 7A) and a NIR wavelength of 750 nm (Fig. 7B). It is noteworthy that the NIR wavelength selected could range from 750 to 800 nm without impacting the NDVI value.

### Comparison of NDVI values from the *NDVIpi* and a commercial NDVI system

**Fig. 8:**
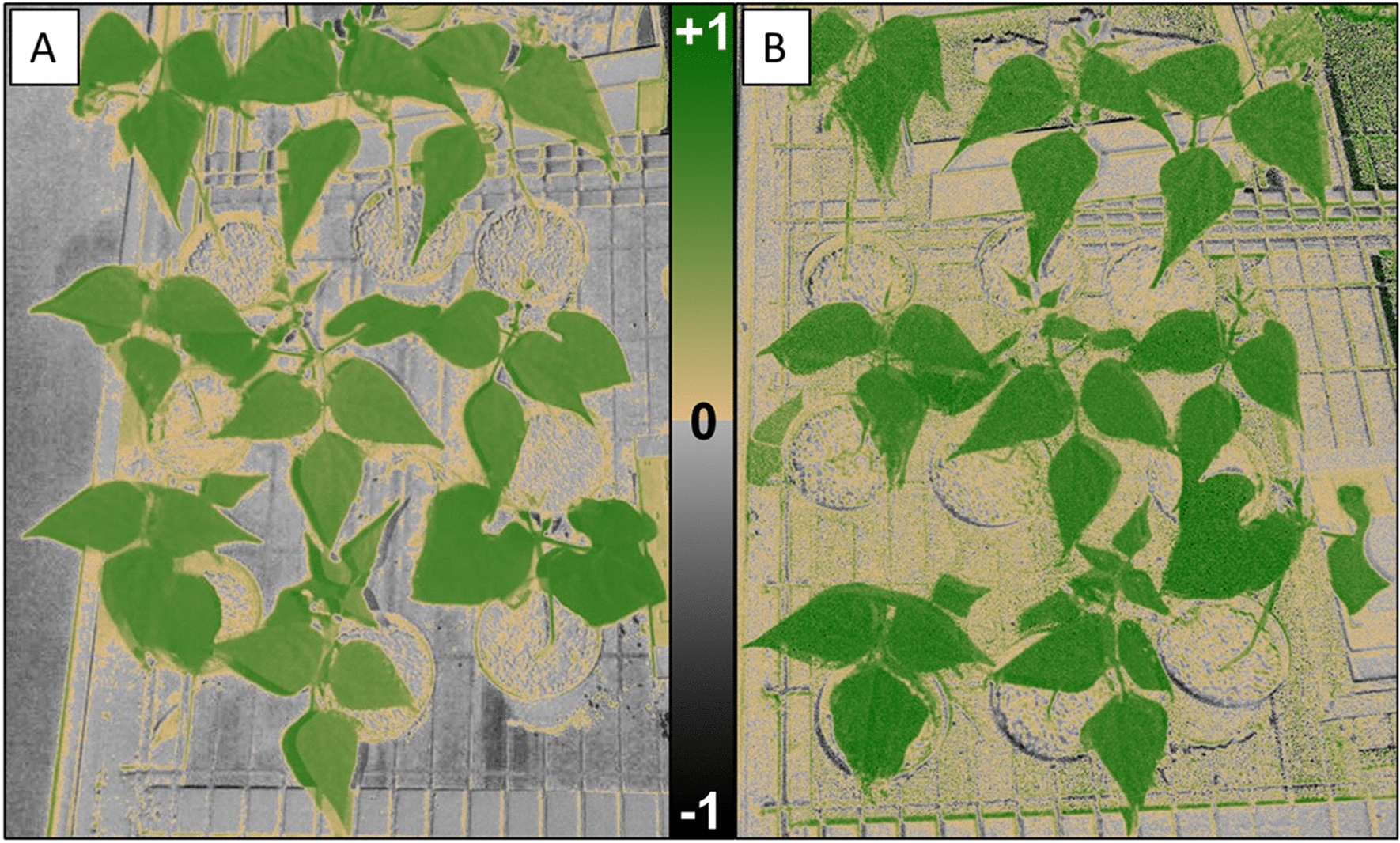
NDVI images of French bean (*Phaseolus vulgaris*), taken in a greenhouse under ambient lighting. **A** NDVI image from the NDVIpi system. **B** NDVI images from the Micasense RedEdge

**Fig. 9:**
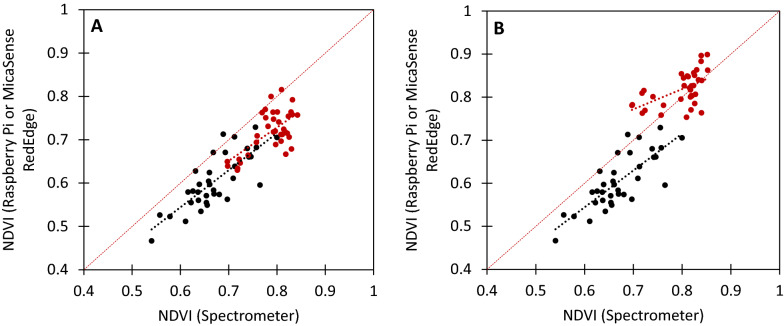
Greenhouse measurements of French bean (*Phaseolus vulgaris*), collected under ambient lighting conditions with nine plants per image. (Black) NDVI measured with the Raspberry Pi, compared to spectrometry NDVI calculated with wavelengths at 620 nm for red and 750 nm for NIR (R^2^ = 0.62, y = 0.86x + 0.03). **A** (Red) NDVI measured by the Micasense RedEdge (NDVI_Micasense1_), compared to spectrometry NDVI calculated with wavelengths at 668 nm for red and 717 nm for NIR (R^2^ = 0.41, y = 0.77x + 0.11). **B** (Red) NDVI measured by the Micasense RedEdge ( NDVI_Micasense2_), compared to spectrometry NDVI calculated with wavelengths at 668 nm for red and 840 nm for NIR (R^2^ = 0.30, y = 0.47x + 0.44)

Images taken using the *NDVIpi* system and a commercially available instrument, Micasense *RedEdge,* were compared and there were clear differences in values between the two cameras (Figs. 8 and 9), which can be attributed to the different wavelengths used by the two systems in the calculation of NDVI (See: Eqs. 2, 4 and 5).

**Fig. 10:**
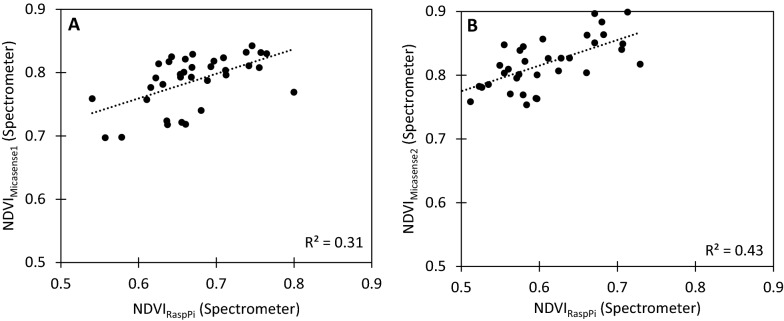
Relationship between an NDVI calculated using NDVI_RaspPi_ and NDVI_Micasense_, as measured by spectrometry. **A** An NDVI calculated using NDVI_RaspPi_ shows a larger range of values before saturation, whereas NDVI_Micasense_ quickly saturates at 0.8. Thus, NDVI_RaspPi_ demonstrates a higher sensitivity to chlorophyll content, and does not saturate easily at high chlorophyll contents. The R^2^ is 0.31, therefore the relationship between the two indices is poor**.** Overall, the Raspberry Pi system shows promise as a robust NDVI imaging system, with higher sensitivity to plant health than common commercial NDVI imaging systems. **B** NDVI_Micasense2_ shows the same effect, however saturation (indicated by values above 0.8) has occurred for all measurements

As each of the camera systems uses different wavelengths to calculate NDVI, this can result in difference in sensitivity and accuracy between instruments. Therefore, a spectrometer was used to measure NDVI with the same wavelengths as the *NDVIpi* (NDVI_RaspPi_) and the Micasense (NDVI_Micasense1_ and NDVI_Micasense2_ indices, Fig. 10), revealing that the NDVI_Micasense_ values may be near saturation for many of the plants, whilst NDVI_RaspPi_ generally had lower values, over a larger range, providing greater differentiation between plants. Calculating the standard deviation between spectrometry NDVI and camera NDVI gives similar values between the two camera systems, with 0.0338 (n = 35, S.D = 0.0185) for the *NDVIpi* and 0.0357 (n = 35, S.D = 0.0175) for the Micasense. The *NDVIpi* system possesses greater detection of differences in NDVI at the lower end of the scale and therefore the *NDVIpi* could be of benefit in discriminating and detecting differences in plant greenness.

## Discussion

Spectral imaging is a widely used research tool for crop monitoring, with popularity increasing in recent years as part of a trend towards greater adoption of precision agriculture. As a result, several low-cost imaging systems have been developed (Table. 1), aimed at increasing availability and uptake, and often re-purposing consumer level digital cameras for spectral imaging [12, 17–25]. Our results have shown the importance in the selection of wavelengths used to calculate NDVI, with a large difference in NDVI estimated between camera systems utilizing different sets of wavelengths. Moreover, we have shown that NDVI measurement accuracy from low-cost systems can be greatly improved by performing a reflectance calibration using multiple reference materials as described here. The methods presented here enable a user to build a NDVI camera system with off-the-shelf equipment that can be calibrated using our approach as described herein and achieve a similar performance to expensive commercially available platforms.

Calibration is an important requirement for all imaging systems to ensure that similar NDVI values are returned regardless of differences in lighting conditions (e.g. changes in light intensity and spectral quality) during image capture. The main advantage of the empirical line method of calibration used here over more sophisticated methods of radiometric calibration [37, 38], is the simplicity and ease of use. However, our method different from many previous methods employing this approach, as we have used 6 references materials to build the model, whilst it is often standard practice to use two. Using only two reference standards assumes a linear relationship between the high and low reflectance standards, and this approach fails to taken into account any gamma corrections within the camera hardware (when present). Here, the increased number of references accounted for the non-linearity of the relationship between the camera signal and the reflectance measurements, including the gamma correction. Alternative calibration protocols have been developed using machine learning algorithms [39] to obtain precise NDVI values. However, these systems still require calibration with a reference standard, and typically need to train the algorithm to adjust to new lighting conditions, camera angles, and therefore are not currently practical for use in the field.

To illustrate the values of increasing the number of references used for image calibration, a comparison between the six references and two references (one with high reflectance and one with low reflectance) shows that while six references has the highest precision, the use of two references still provides a reasonable calibration (Additional file 1: Fig. S5). Two reference standards may be beneficial in situations in which the use of six references may be impractical due to technical or operational limitations. For instance, it is common for UAV imagery to capture an image of an in situ white reference prior to field imagery (or re-imaged when lighting conditions change), using the pixel value for the white reference to calculate reflectance in all subsequent imagery (by assuming 0% reflectance is represented by a pixel value of 0).

Likewise, in situations in which red reflectance is very low (e.g. dense canopy, very dark green plants), camera settings (i.e. shutter speed, exposure, etc.) can be adjusted so subsequent visible red images can be calibrated to a reference that has lower reflectance (e.g. 30%), allowing for the full range of camera to be used for this lower range of reflectance values, which can increase the ability of the system to measure smaller variations of NDVI.

The *NDVIpi* uses two cameras to prevent NIR light from contributing to red images, as a single camera based system will have NIR leaking unless high quality optics are used, creating biases in the estimation of NDVI. On the other hand, in a two camera system, the NIR camera does measure a small amount of red light that is able to pass through the filter, as mentioned previously (Additional file 1: Figs. S1, S2). Additionally, both camera types will also measure some amount of blue and green irradiance on the red colour channel, which is likely to add some degree variation to calibrated images and the resulting NDVI measurements. However, this effect is likely to be small, demonstrated by the strong relationship between *NDVIpi* camera measurements and spectrometer measurements (Fig. 6).

The use of two cameras can create issues during image alignment [20, 22], with images misalignment the closer the camera is to a plant when it is not flat and horizontal relative to the camera (for example, tall grasses)*.* This is less of an issue the further the camera is from the subject of the image, due to a reduced parallax with increasing distance. Thus, for instance, images collected by UAV are vastly less susceptible to alignment issues than images collected from a handheld or land vehicle mounted device. However, the benefits of a two camera system, as well as the most common usage for an NDVI camera being UAV imagery, far outweigh any potential issues with image alignment, and thus the majority of commercial devices employ multi-camera systems, including the Micasense RedEdge used here. Some systems, such as that by Anika et al. [22], overcome the physical limitations of multi-camera setups by utilizing a single camera capable of recording both visible and NIR imagery simultaneously, and using algorithms to extract separate visible and NIR imagery. Likewise, a camera with a filter wheel could also be used to similar effect, providing the filters were of high enough quality to ensure NIR is fully blocked from leaking onto visible red images.

### Comparison of *NDVIpi* imagery against a commercial NDVI system

Images captured using both the *NDVIpi* and commercial *Micasense RedEdge* were lower in NDVI values than measurements collected using the spectrometer (Fig. 9). Likewise, an offset was also obvious in the relationship between NDVI values collected with a spectrometer and those from the *NDVIpi* on French bean (Fig. 6A). Analysis of image skewness (Additional file 1: Fig. S4) showed that French Bean NDVI values were lower when measured by the spectrometer compared to the camera system, which could have been due to differences in the measuring area. The spectrometer was used with a leaf clip and measurements were collected from a relatively small area of each quadrant of each leaf measured. The *NDVIpi* on the other hand, measured the entire leaf area present within a single image. As a result, structural areas with lower NDVI values such as veins, could had a stronger influence on NDVI measured with the leaf clip compared to imaging methods that considered the entire leaf.

There was a clear difference in NDVI values collected using the *NDVIpi* system and the commercial Micasense RedEdge, with values collected using the Micasense RedEdge higher (0.70–0.90) than the *NDVIpi* (0.54–0.80) (Figs. 9A, 10A). This was attributed to the different wavelengths used in the two systems to measure visible and far-red bands. The red wavelength at 665 nm used by the Micasense (NDVI_Micasense1,_ Eq. 4) is close to the chlorophyll absorption peak at 680 nm [15], whereas the shorter wavelength at 620 nm used by the *NDVIpi* (NDVI_RaspPi_, Eq. 2) further from the absorption peak. The further away red wavelengths are from the absorption peak (i.e. between 600 and 680 nm), the more sensitive they are to changes in reflection (Additional file 1: Fig. S6). Likewise, the closer the red wavelength is to 680 nm, the higher the resulting NDVI value will be due to increased absorption of that wavelength by chlorophyll, especially so with higher chlorophyll content. This suggests that the NDVI_Micasense1_ and NDVI_Micasense2_ may saturate when measuring plants with high chlorophyll content. A different selection of wavelengths can thus alter the ratio of red to NIR based on the % reflection of the wavelength used, which varies across the red spectral region (i.e. 600–700 nm, see: Additional file 1: Fig. S6) and thus the output of the two systems not directly comparable, although in general NDVI for vegetation saturates at values from 0.8 to 0.9 for healthy vegetation [40].

While there are indications that the NDVI_RaspPi_ determined by the *NDVIpi* is more sensitive to smaller changes in plant greenness, it should be noted that the broadband channels used by the *NDVIpi* can be susceptible to changes in reflected light from other wavelengths, within both the red or NIR spectrum. The apparent relatively lower sensitivity to chlorophyll content of the NDVI_Micasense1_ and NDVI_Micasense2_ (Eqs. 4, 5) from the Micasense RedEdge may be more desirable in agricultural applications when for example when crops areas [41–43], as the systems will be better at differentiating vegetation and non-vegetation material. However, a system with higher sensitivity to chlorophyll but overall lower NDVI values, such as the one we have developed here is capable of discriminating between species or cultivars, and therefore beneficial in screening for breeding [44].

### Comparison of *NDVIpi* with commercial NDVI imaging systems

Using the *NDVIpi* imaging system has many advantages, such as ease of use, repairability, and lower price point due to the use of open source and off-the-shelf components, which in the case of a fault or breakage, can be fixed or replaced by the user relatively easily. In total, the system (including power source, screen, etc.) can be procured for approximately US$400–US$500. The *Micasense RedEdge* for instance, retails at approximately US$4500–US$5000 at the time of publishing but does have extra features such as integrated GPS and built-in wireless control. Other systems include the customisable and modular Tetracam *Macaw 6*, retailing at US$14,000 with the ability to swap optical filters, or the smaller *Tau2* for US$8000 per camera which measures green, red and NIR imagery, and down-welling irradiance for reflectance calibration which negates the need to use external targets within the image. Other companies such as *Max Max*, modify commercial cameras specifically for NDVI imagery, with optical filters being added. The price range for these systems can range from US$1600 up to US$6000. Imaging system for NDVI can also be built using consumer level camera equipment, through the removal of the IR filter [18] and in its place a new filter that blocks all light below NIR wavelengths (e.g. 710 nm) installed. Overall, the performance price ratio is in favour of the *NDVIpi* imaging system as it has a similar performance to commercially available systems for a fraction of the price. Other NDVI systems have been developed utilizing off-the-shelf components but use more expensive optical filters to obtain narrowband measurements [45] and/or downwelling spectral measurements from a spectrometer, which increases the expensive of these systems [46] (Table 1).

As far as the authors are aware, our methodology is the first to use the Raspberry Pi platform to develop a low-cost dual-camera NDVI, and use a 6 standard calibration approach. Our system is one of the few that does not use conventional optical filters, opting for low-cost colour lighting filters instead. This system was also developed to use customisable open source software for image calibration and alignment. All features of the NDVIpi confer a significant advantage in reducing price, accessibility, and customisation that currently is not widely available.

The Raspberry Pi itself is, by design, easy to use and customisable, and thus a major advantage of the system is easy integration into any new or pre-existing applications—e.g. mounted in greenhouses, or on farm vehicles and UAVs to capture imagery of entire fields. For research applications this also allows the system to be integrated with other imaging based systems. For instance, thermography, another imaging based technique, is used to measure evapotranspiration and stomatal conductance of plants [48–52], and thus also used to indicate water status [53, 54]. Currently, thermography enabled devices are rapidly decreasing in both size and price, and therefore would be a logical next step in integrating with NDVI imagery. NDVI imaging is regularly used agriculturally as an indicator of chlorophyll and nitrogen content to optimise fertilizer applications. A combined NDVI and thermal imaging system could give an overview of the two primary factors of crop performance, water status and nitrogen content, at an affordable price compared to many multispectral or multi-technique devices, while being more accessible.

Finally, the NDVIpi can be repurposed to image other spectral indices. For instance, suitable optical or colour filters could be utilised to capture desired visible and/or NIR spectral regions for a number of other spectral indices related to, e.g.; water content [55], chlorophyll or anthocyanin content [56, 57], and the status of the xanthophyl cycle [58].

## Conclusion

Here we present a methodology for the measurement of NDVI that utilizes low cost, off-the-shelf components, (two Raspberry Pi cameras and a Raspberry Pi Compute module) without compromising accuracy. To achieve high accuracy a calibration method using six calibration targets of known spectra reflectance was developed to convert collected red and near-infrared images into images of spectral reflectance. Moreover, our results highlight the importance of the choice of red wavelengths in the calculation of NDVI, which resulted in differences in accuracy and sensitivity between camera systems. The *NDVIpi* imaging system was proven to be a robust tool for the measurement of NDVI, comparable to other commercial systems but for a fraction of the cost, and thus it can be used to reliably and accurately measure plant health. Overall, the *NDVIpi* system produces comparable NDVI measurements to ‘gold-standard’ spectrometry measurements and NDVI imagery with a popular commercial camera, the Micasense RedEdge. The *NDVIpi* also showed a higher sensitivity to chlorophyll content than NDVI from the Micasense RedEdge, due to the choice of red wavelength, but at a cost of greater variation. However, the Micasense RedEdge could be considered more beneficial for situations in which the price of the system is not a concern, or when indices other than NDVI (e.g. NDRE) are required which is an important consideration.

## Supplementary Information

**Additional file 1:** **Fig. S1** Transmission of light (as a percentage) across the visible spectrum (400nm to 700nm) for the filter Alice Blue 197 (LEE Filters). Strong transmission occurs in the blue regions (from 400nm to 500nm), and weak (<15%) transmission in the red regions. The filter allows NIR (>700nm) to transmit through the filter without much loss (>80% transmittance). **Fig. S2** (A) Simulated light levels detected by a camera lens from a simulated 800 µmol m^−2^ s^−1^ light source, reflected from modelled leaf reflectance for leaves with varying chlorophyll content, and measured after transmission through the Alice Blue filter. The measured NIR light, with added red light representing the 5%-15% transmission of red light through the Alice Blue filter, is compared to PPFD measured with a simulated camera lens that only receives NIR light, without the extra transmission of red light. ● represents the PPFD of measured NIR, if red light leaking onto the red channel was zero (1:1 relationship for NIR). ○ represents the PPFD of both NIR and additional red light, as transmitted through the filter. The distance between the two measurements is the amount of visible red light that is transmitted through the filter and is therefore measured as additional NIR light by the camera, which was calculated as 0.92955% at 800 μmol m^-2^ s^-1^. (B) Simulated NDVI calculated from same dataset, showing the NDVI for a camera with no red light transmission by the Alice Bue filter, and NDVI with some red light transmission. The increase in red light on the NIR channel is seen here by the overestimation of NDVI at low values. More details on the simulated leaf reflectance can be found in Fig. S6. **Fig. S3** Reflectance of six Kayospruce Odyssey materials. White (#1), Sand (#2), Brown (#3), Indian Burch (#4), Forest Green (#5), Burgundy (#6), measured with a spectrometer. These materials were chosen for their relative uniformity across the red and near infra-red spectrum, and to ensure a range across reflectance values. **Fig. S4** Skewness of NDVI measurements taken by the NDVIpi in 40 selected images of French Bean (Phaseolus) and Wheat, showing a greater skew towards a lower NDVI for the broad leaf Phaseolus (Skewness = 1.16, S.D. = 0.49) compared with grass leaves (Skewness = 0.67, S.D. = 0.47). Skewness was calculated based on the histogram of each leaf that was the target of measurement within each image. **Fig. S5** Comparison of images calibrated with either **(A) **Two or **(B)** Six references, against NDVI calculated using spectrometry data for wheat and barley. Using six calibration references may be suitable in a laboratory or in low throughput phenotyping; however for taking multiple images in the field, such as with a UAV based setup for crop imaging, the use of six references will often be unpractical. Therefore, images of wheat and barley were also calculated using only two references; the highest reflecting material (white), and the lowest reflecting material (green). When calibrating images with only two reference standard, image digital numbers were transformed to eliminate the effect of gamma correction, which would otherwise introduce inaccuracy in calibration when using just two references. The de-gamma process was performed by transforming the digital number of each pixel by the power of 2.12766, a value that was determined empirically. **Fig. S6** Simulated changes in reflectance due to changes in chlorophyll a+b, modelled using the PROSAIL model across simulated chlorophyll a+b content from 0 µg cm^-2^ (bottom grey line) up to 60 µg cm ^-2^ (top blue line) at 5 µg cm^-2^ intervals. The entire region from 600nm up to 680nm shows sensitivity to chlorophyll content. Thus, calibration using any wavelength with the red (600nm up to 680nm) spectrum will be sensitive to chlorophyll content, however the further away the wavelength is from the chlorophyll absorption peak at 680nm, the greater the associated change in reflectance and thus increased sensitivity to chlorophyll content. Highlighted areas correspond to the wavelengths (620nm and 750nm) used by the Raspberry Pi system. Simulated reflectance data was obtained by generating a series of representative reflectance spectra with varied levels of reflectance in the NIR and visible red spectra, corresponding to theoretical changes in chlorophyll content, by using the PROSAIL [66] leaf reflectance model. The model works by using inputs of leaf anatomy, such as leaf thickness and chlorophyll content, and considers the leaf as consisting of multiple ‘layers’ (e.g. layers representing leaf thickness, water content, pigments, etc.). These layers are treated as semi-transparent plates, and total reflection, refraction and transmission for each plate is calculated. Similarly, scattering and absorption of each plate are also calculated. The sum of all plates yields the total reflection and transmission of light through the modelled leaf. Increasing or decreasing the layers as defined by the input parameters affects the interaction between irradiance, the absorption of light by pigments, and refraction due to the physical structure of the leaf, thus simulating the total percentage of light which is reflected and transmitted. Simulated reflectance was calculated with varying levels of chlorophyll a+b concentration from 0 µg cm^−3^ up to 60 µg cm^−3^, at 5 µg cm-3 intervals (See Supplementary S3), resulting in an output of 13 simulated leaves. The remaining inputs were set to; Leaf structure, 1.2; Carotenoid content, 10 µg/cm²; Brown pigments, 1.0; Equivalent water thickness, 0.015 cm; Leaf mass per unit area, 0.009 g/cm².

## Data Availability

The datasets used and/or analysed during the current study are available from the corresponding author on reasonable request.
