# Supplementary material for: Development of an accurate low cost NDVI imaging system for assessing plant health
Source: Plant Methods. 2023 Jan 30;19:9. doi: 10.1186/s13007-023-00981-8 (PMC9887843; doi:10.1186/s13007-023-00981-8)

**Additional file**

**Figure. S1** Transmission of light (as a percentage) across the visible spectrum (400nm to 700nm) for the filter Alice Blue 197 (LEE Filters). Strong transmission occurs in the blue regions (from 400nm to 500nm), and weak (<15%) transmission in the red regions. The filter allows NIR (>700nm) to transmit through the filter without much loss (>80% transmittance).


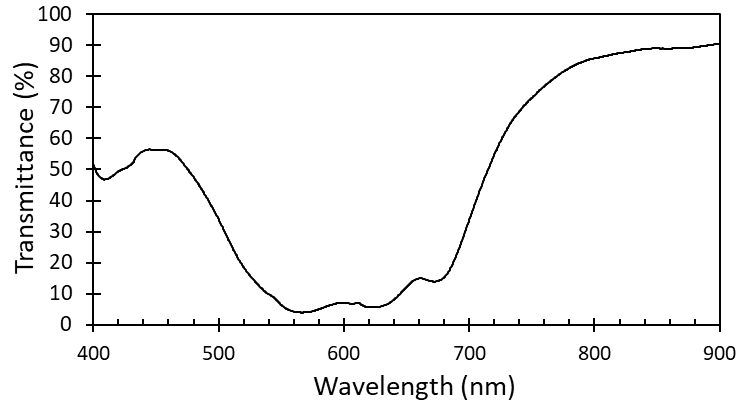

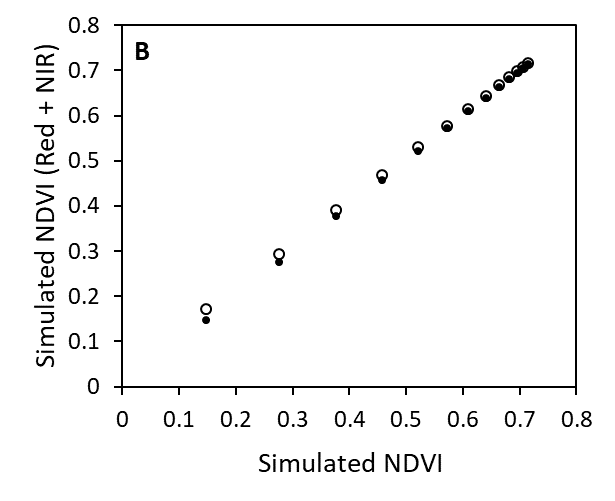

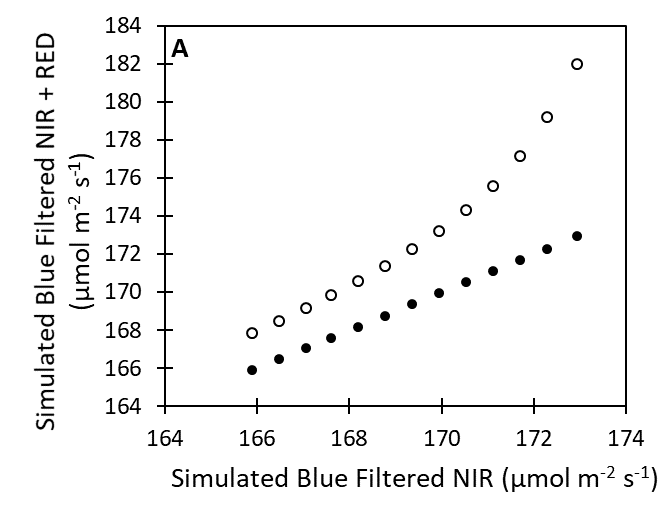


**Figure. S2 (A)** Simulated light levels detected by a camera lens from a simulated 800 µmol m^−2^ s^−1^ light source, reflected from modelled leaf reflectance for leaves with varying chlorophyll content, and measured after transmission through the Alice Blue filter. The measured NIR light, with added red light representing the 5%-15% transmission of red light through the Alice Blue filter, is compared to PPFD measured with a simulated camera lens that only receives NIR light, without the extra transmission of red light. ● represents the PPFD of measured NIR, if red light leaking onto the red channel was zero (1:1 relationship for NIR). ○ represents the PPFD of both NIR and additional red light, as transmitted through the filter. The distance between the two measurements is the amount of visible red light that is transmitted through the filter and is therefore measured as additional NIR light by the camera, which was calculated as 0.92955% at 800 μmol m^-2^ s^-1^

**(B)** Simulated NDVI calculated from same dataset, showing the NDVI for a camera with no red light transmission by the Alice Bue filter, and NDVI with some red light transmission. The increase in red light on the NIR channel is seen here by the overestimation of NDVI at low values.

More details on the simulated leaf reflectance can be found in Supplementary S6.

**Figure. S3** Reflectance of six Kayospruce Odyssey materials. White (#1), Sand (#2), Brown (#3), Indian Burch (#4), Forest Green (#5), Burgundy (#6), measured with a spectrometer. These materials were chosen for their relative uniformity across the red and near infra-red spectrum, and to ensure a range across reflectance values.


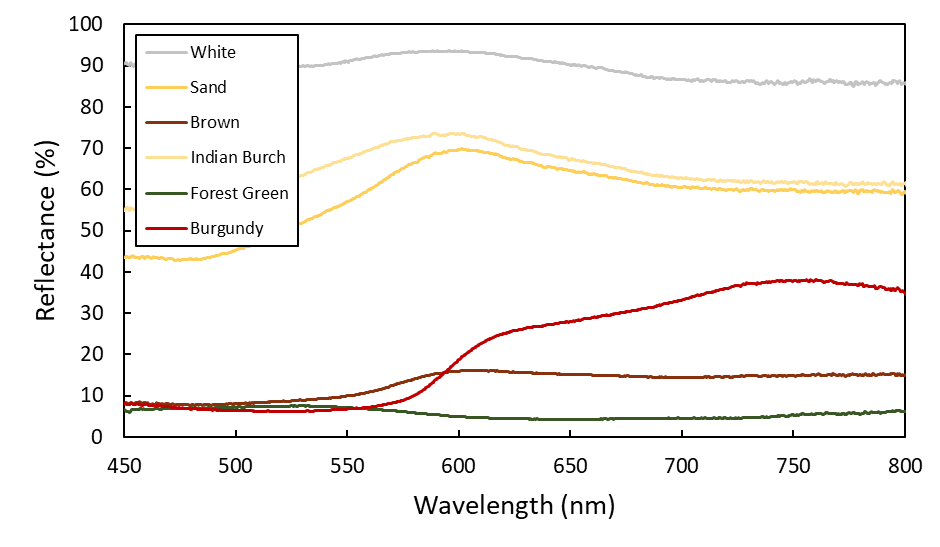


**Figure. S4** Skewness of NDVI measurements taken by the NDVIpi in 40 selected images of French Bean (Phaseolus) and Wheat, showing a greater skew towards a lower NDVI for the broad leaf Phaseolus (Skewness = 1.16, S.D. = 0.49) compared with grass leaves (Skewness = 0.67, S.D. = 0.47). Skewness was calculated based on the histogram of each leaf that was the target of measurement within each image.


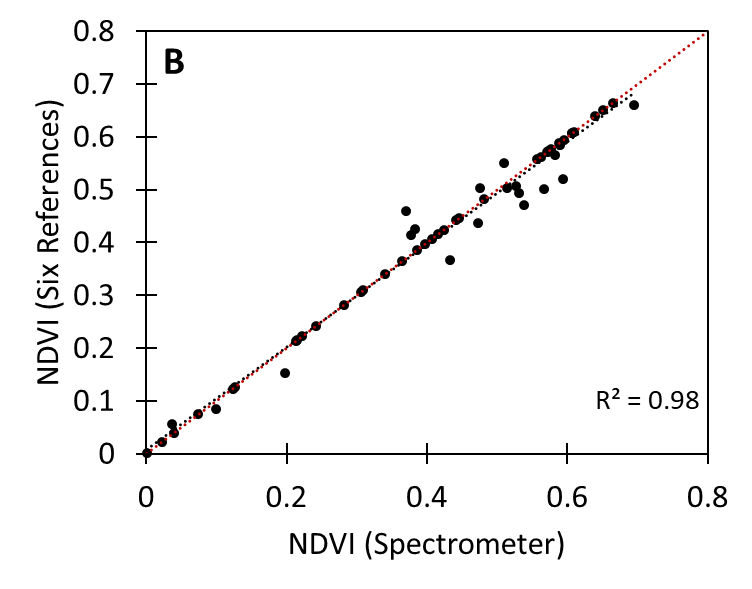

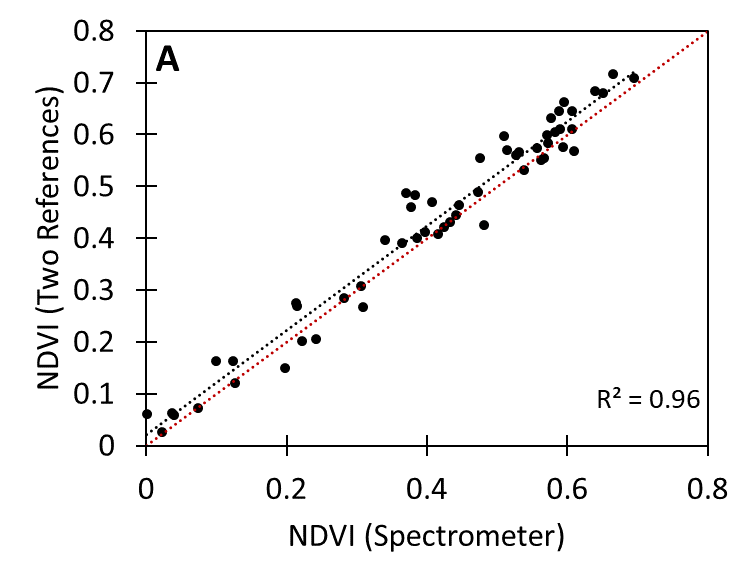


**Figure. S5** Comparison of images calibrated with either **(A)** Two or **(B)** Six references, against NDVI calculated using spectrometry data for wheat and barley. Using six calibration references may be suitable in a laboratory or in low throughput phenotyping; however for taking multiple images in the field, such as with a UAV based setup for crop imaging, the use of six references will often be unpractical. Therefore, images of wheat and barley were also calculated using only two references; the highest reflecting material (white), and the lowest reflecting material (green). When calibrating images with only two reference standard, image digital numbers were transformed to eliminate the effect of gamma correction, which would otherwise introduce inaccuracy in calibration when using just two references. The de-gamma process was performed by transforming the digital number of each pixel by the power of 2.12766, a value that was determined empirically.

The use of two or six references in the calibration process was assessed to determine the impact on NDVI values. Using only two references produces NDVI measurements that are less precise, yielding a weaker relationship between NDVIpi and spectrometer (R2 = 0.96, Supplementary Figure S9A) to spectrometry data than six references (R2 = 0.98, Supplementary Figure S9B). The range of NDVI values from using two references for calibration also demonstrated slightly higher overall NDVI values (Supplementary S9 A).

**Figure. sS6** Simulated changes in reflectance due to changes in chlorophyll a+b, modelled using the PROSAIL model across simulated chlorophyll a+b content from 0 µg cm ^-2^ (bottom grey line) up to 60 µg cm ^-2^ (top blue line) at 5 µg cm^-2^ intervals. The entire region from 600nm up to 680nm shows sensitivity to chlorophyll content. Thus, calibration using any wavelength with the red (600nm up to 680nm) spectrum will be sensitive to chlorophyll content, however the further away the wavelength is from the chlorophyll absorption peak at 680nm, the greater the associated change in reflectance and thus increased sensitivity to chlorophyll content. Highlighted areas correspond to the wavelengths (620nm and 750nm) used by the Raspberry Pi system.

Simulated reflectance data was obtained by generating a series of representative reflectance spectra with varied levels of reflectance in the NIR and visible red spectra, corresponding to theoretical changes in chlorophyll content, by using the PROSAIL [69] leaf reflectance model. The model works by using inputs of leaf anatomy, such as leaf thickness and chlorophyll content, and considers the leaf as consisting of multiple ‘layers’ (e.g. layers representing leaf thickness, water content, pigments, etc.). These layers are treated as semi-transparent plates, and total reflection, refraction and transmission for each plate is calculated. Similarly, scattering and absorption of each plate are also calculated. The sum of all plates yields the total reflection and transmission of light through the modelled leaf. Increasing or decreasing the layers as defined by the input parameters affects the interaction between irradiance, the absorption of light by pigments, and refraction due to the physical structure of the leaf, thus simulating the total percentage of light which is reflected and transmitted. Simulated reflectance was calculated with varying levels of chlorophyll a+b concentration from 0 µg cm-3 up to 60 µg cm-3, at 5 µg cm-3 intervals (See Supplementary S3), resulting in an output of 13 simulated leaves. The remaining inputs were set to; Leaf structure, 1.2; Carotenoid content, 10 µg/cm²; Brown pigments, 1.0; Equivalent water thickness, 0.015 cm; Leaf mass per unit area, 0.009 g/cm².


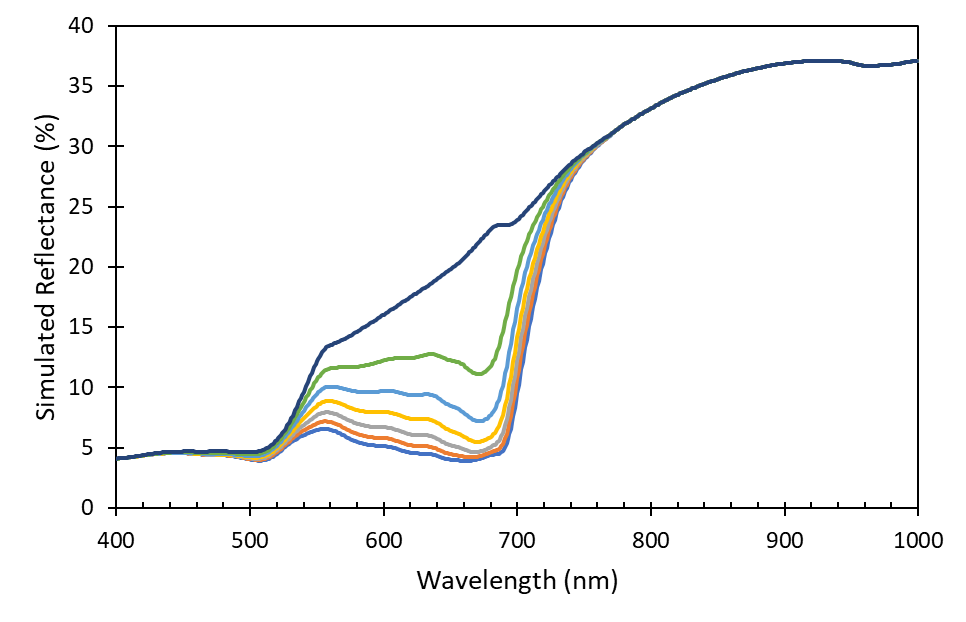

Supplement: Supplementary file 1 — Additional file 1: Fig. S1 Transmission of light (as a percentage) across the visible spectrum (400nm to 700nm) for the filter Alice Blue 197 (LEE Filters). Strong transmission occurs in the blue regions (from 400nm to 500nm), and weak (<15%) transmission in the red regions. The filter allows NIR (>700nm) to transmit through the filter without much loss (>80% transmittance). Fig. S2 (A) Simulated light levels detected by a camera lens from a simulated 800 µmol m−2 s−1 light source, reflected from modelled leaf reflectance for leaves with varying chlorophyll content, and measured after transmission through the Alice Blue filter. The measured NIR light, with added red light representing the 5%-15% transmission of red light through the Alice Blue filter, is compared to PPFD measured with a simulated camera lens that only receives NIR light, without the extra transmission of red light. ● represents the PPFD of measured NIR, if red light leaking onto the red channel was zero (1:1 relationship for NIR). ○ represents the PPFD of both NIR and additional red light, as transmitted through the filter. The distance between the two measurements is the amount of visible red light that is transmitted through the filter and is therefore measured as additional NIR light by the camera, which was calculated as 0.92955% at 800 μmol m-2 s-1. (B) Simulated NDVI calculated from same dataset, showing the NDVI for a camera with no red light transmission by the Alice Bue filter, and NDVI with some red light transmission. The increase in red light on the NIR channel is seen here by the overestimation of NDVI at low values. More details on the simulated leaf reflectance can be found in Fig. S6. Fig. S3 Reflectance of six Kayospruce Odyssey materials. White (#1), Sand (#2), Brown (#3), Indian Burch (#4), Forest Green (#5), Burgundy (#6), measured with a spectrometer. These materials were chosen for their relative uniformity across the red and near infra-red spectrum, and to ensure a range acros [file 13007_2023_981_MOESM1_ESM.docx]
